# The p48 MW flow modulation device for treatment of unruptured, saccular intracranial aneurysms: a single center experience from 77 consecutive aneurysms

**DOI:** 10.1186/s42155-020-00131-4

**Published:** 2020-08-09

**Authors:** Muhammad AlMatter, Elina Henkes, Alexander Sirakov, Marta Aguilar Pérez, Victoria Hellstern, Carmen Serna Candel, Oliver Ganslandt, Hans Henkes

**Affiliations:** 1 Neuroradiologische Klinik, Klinikum Stuttgart, Kriegsbergstraße 60, 70174 Stuttgart, Germany; 2 Radiology Department, UH St Ivan Rilski, Sofia, Bulgaria; 3 Neurochirurgische Klinik, Klinikum Stuttgart, Stuttgart, Germany; 4 Medizinische Fakultät der Universität Duisburg-Essen, Essen, Germany

**Keywords:** Aneurysm, Flow diversion, New device, p48 MW, Endovascular treatment

## Abstract

**Background:**

The p48 MW Flow Modulation Device (phenox, Bochum Germany) is a low profile flow diverter stent (FDS), designed for implantation into intracranial arteries with a diameter of less than 3.5 mm.

**Objective:**

To evaluate the safety and efficacy of the p48 MW FDS in the treatment of unruptured aneurysms located at intracranial arteries with less than 3.5 mm diameter based on a retrospective analysis from a single tertiary neurovascular center.

**Methods:**

A prospectively maintained database was retrospectively reviewed to identify all cases of intracranial saccular aneurysms treated electively with the p48 MW device. Records were made of basic demographics, aneurysmal characteristics, interventional procedures, adverse events, clinical outcomes and occlusion rates on angiographic follow-ups.

**Results:**

A total of 77 aneurysms and 74 patients were included. The mean size of the treated aneurysms was 3.5 ± 2.4 mm and the mean aspect ratio was 1.3 ± 0.4. A total of 80 endovascular procedures were performed with a total of 12 (15%) adverse events leading to two (2.5%) permanent morbidities/mortalities. Technical issues were encountered in 3 (3.9%) cases. Adequate occlusion of the treated aneurysm was recorded in 55.6% and 63.9% on the first and latest available DSA follow-ups, respectively. There were no cases of side-branch occlusion.

**Conclusions:**

The p48 MW is an easy-to-use implant with very good safety margins. Side branch occlusion and significant in-stent stenosis are infrequently encountered. The time from implantation to sufficient aneurysm occlusion takes longer than with FDS with lower porosity.

## Introduction

Flow diverter stents (FDS) are now important tools in the neurointerventional armamentarium expanding the spectrum of treatable aneurysms by endovascular means. Although initially developed for the treatment of side-wall aneurysms of the internal carotid artery (ICA), their use has been increasingly expanding to include lesions involving smaller and more distal vessels, bifurcation aneurysms and also for treatment of challenging pathologies such as dissecting, fusiform, and blood-blister aneurysms (Puri et al., 2016; Cagnazzo et al., 2019; Bhogal et al., 2018; Saleme et al., 2014; Gawlitza et al., 2016; Rouchaud et al., 2015; Bhogal et al., 2017; Limbucci et al., 2020). This expansion of the spectrum of treatable lesions was invariably associated with the need for technical improvement of FDS such as better navigability, lower profile and the compatibility with small microcatheters to enable better and safer distal navigation. The p48 MW Flow Modulation Device (phenox, Bochum, Germany) is a newer generation FDS intended for the use in smaller vessels of less than 3.5 mm diameter and can be deployed through a 0.021″ inner diameter microcatheter. We report our initial experience with this device in the treatment of unruptured, saccular intracranial aneurysms with emphasis on technical feasibility, efficacy and procedural complication based on 77 consecutive aneurysms from a single tertiary neurovascular center.

## Methods

### Study population

The prospectively maintained institutional database was retrospectively reviewed using the following criteria: patients who were treated for unruptured (or not acutely ruptured) intradural, saccular aneurysms using the p48 MW Flow Modulation Device as the main treatment strategy. Patients who were treated for a recurrence or a significant remnant after endovascular coiling or surgical clipping were also included. Patients who were previously treated using an endoluminal device in the parent vessel were excluded as a preexisting stent may reduce the efficacy of FDS (Shapiro et al., 2017). Records were made of basic demographic data, characteristics of the treated aneurysms including any previous treatments of the target lesion.

### Device description

The p48 MW Flow Modulation Device is a tubular vascular implant that consists of 48 interwoven nitinol wires which are filled with a platinum core to ensure visibility under X-ray fluoroscopy. The delivery system has a platinum marker at the distal end of the transport tube and another one at the distal wire tip to allow the operator to determine its position under fluoroscopy. The attachment of the implant to the delivery system follows the “friction locking principle”. The proximal end of the implant is secured between a soft polymer pad at the distal end of the transport tube and an introducer sheath (after removal of the sheath, the function is performed by the microcatheter), in such a way that pushing and pulling of the implant is enabled. The product is stored in an introducer sheath and is transferred into a microcatheter with an inner diameter of 0.021″ (0.53 mm). This sheath is moved proximally during insertion of p48 MW to enable complete passage through the microcatheter. The implant self-expands as it leaves the microcatheter. The device can be completely re-sheathed, repositioned or removed up to the point of maximum implant deployment indicated by a platinum marker at the distal end of the transport tube. The p48 MW is deployed by means of a coordinated movement, whereby the microcatheter is withdrawn and the delivery system is advanced to avoid any movement of the distal implant end by the shortening effect. Due to the foreshortening effect, the distal delivery wire tip moves distally during deployment. To counteract this movement in order to avoid, e.g., the entry of the delivery wire tip into distal sensitive vessels, the delivery wire tip can be moved proximally. After final control of deployment and position, the implant is completely deployed and detached from the delivery system by withdrawal of the microcatheter.

### Endovascular procedures

The indication for the endovascular treatment was decided in each case on an individual basis after extensive consultation of the patients or their legal representatives on the disease, treatment alternatives and possible risks and complications. All patients were pretreated with dual antiplatelet therapy (DAPT) at least 48 h prior to the scheduled procedure. The platelet function response was tested using both the Multiplate Analyzer (Roche Diagnostics, Mannheim, Germany) and the VerifyNow Analyzer (Accumetrics, San Diego, CA) and the dosage/choice of antiplatelet agent were modified if needed in order to achieve significant dual inhibition. All endovascular procedures were performed under general anesthesia using dedicated biplane DSA systems (Axiom, Siemens, Erlangen, Germany). All procedures were performed by one of two senior interventional neuroradiologists with a vast experience in the use of FDS. The interventions were carried out via a right femoral artery approach using a short introducer sheath. A standard bolus of 3000 IU of non-fractionated heparin was given intravenously after securing the sheath. The cervical segment of the ICA or the vertebral artery (VA) was accessed with a 6F guiding catheter or a combination of an 8F guiding catheter and an intermediate catheter according to the operator’s preference and vessel-tortuosity. Navigation of the target vessel was done with a 0.021″ microcatheter (Prowler Select Plus, Codman; Trevo Pro 18, Stryker) over a standard length 0.014″ microguidewire (Synchro2, Stryker; pORTAL, phenox) under roadmap guidance. In cases of difficult distal-vessel navigation due to very acute artery angulation, a steam shaped low-profile microcatheter with a tight curve was first navigated to the target vessel and then exchanged for the 0.021″ microcatheter over an exchange-length microwire. The p48 MW device was then inserted into the microcatheter and navigated to the level of the aneurysm. Deployment was carried out by active unsheathing of the device by retracting the microcatheter for the first third and then passive unsheathing by pushing on the delivery wire for appropriate expansion of the struts and good wall apposition. The DAPT was maintained for 1 year after treatment and then switched to monotherapy with aspirin indefinitely.

### Clinical and angiographic follow-ups

As per our institutional routine, the first follow-up with digital subtraction angiography (DSA) combined with clinical evaluation were performed 6 months after the treatment. A second DSA follow-up was usually performed after 1 year. Extended follow-ups thereafter were decided on an individual basis. Extended follow-ups were performed in cases with incomplete occlusion of the aneurysm or in cases of morphological changes to the parent vessel. Assessment of the aneurysmal occlusion after flow diversion was recorded in a simplified fashion as either complete occlusion, neck remnant filling or persistent aneurysm perfusion. A cranial magnetic resonance imaging (cMRI) and clinical assessment were performed upon discharge after treatment and upon each radiological follow-up if considered necessary. The neurological assessments were performed by a neurologist or a certified stroke nurse and recorded using the modified Rankin Scale (mRS).

### Outcome measures

The primary aim of this study was to assess the safety and efficacy of the p48 MW Flow Modulation Device for the treatment of unruptured aneurysms. Records were therefore made of all generic and procedure-related complications, both temporary and permanent and both symptomatic and asymptomatic. As aneurysmal occlusion after flow diversion is a dynamic process, the latest available DSA follow-up was used to assess the efficacy of the treatment.

## Results

The institutional database for endovascular treatments with the p48 MW device consisted of 89 patients and 92 aneurysms treated with a total of 100 p48 MW devices between November 2016 and September 2019. A total of 77 aneurysms in 74 patients met the aforementioned inclusion criterias and constituted the cohort of this report. The remaining 15 patients were excluded due to acute rupture status (2 cases), fusiform or dissecting morphology of the treated aneurysm (8 cases) or pre-existing endoluminal device (5 cases).

### Patients and aneurysms

The mean age of the included patients at the time of treatment was 57 ± 11.4 years ranging from 29.5–83.4 years. Females made up the majority of the study population with 83.8%. Eleven patients (14.9%) were previously treated with plain coiling in the acute rupture state and with the p48 MW device in a staged second intervention while 13 (17.6%) cases were previously treated due to a ruptured aneurysm at a different location. In the remaining 67.5% the treated aneurysms were found incidentally. The initial clinical status was scored as mRS 3, 2, 1 and 0 in1 (1.3%), 7 (9.5%), 17 (23%) and 49 (66.2%) patients, respectively. As stated in the methods section, only saccular, not acutely ruptured aneurysms were included. The mean size of the 77 aneurysms was 3.5 ± 2.4 mm (median 3 mm, range 1–17 mm) and the mean neck width was 2.7 ± 1.3 mm (median 2 mm, range 1–7 mm) with a mean aspect ratio of 1.3 ± 0.4 (median 1.2, range 0.5–2.5). Table 1 summarizes the location and size of the treated aneurysms in this series.

**Table 1:** Location and size of the 77 aneurysms included in this series

Location	Nr.(%)	Fundus (in mm)	Neck (in mm)	Aspect ratio
ICA	4 (5.2)	5.8	3.8	1.6
PcomA	2 (2.6)	5	4	1.7
ICA (misc.)	2 (2.6)	6.5	3.5	1.6
ACA	39 (50.6)	3.7	2.7	1.6
A1	4 (5.2)	6.3	3	1.9
A1/A2	7 (9.1)	1.9	1.7	1
AcomA	20 (26)	4.2	3.2	1.3
Pericallosal	8 (10.4)	3	2	1.4
MCA	22 (28.6)	3.2	2.5	1.3
M1	21 (27.3)	3.2	2.5	1.3
M2	1 (1.3)	3	2	1.5
Basilar artery	2 (2.6)	2.5	1.8	1.5
PCA	5 (6.5)	2	2.6	0.8
SCA	4 (5.2)	1.5	1.8	0.9
PICA	1 (1.3)	9	6	1.5

*Abb.*: *ICA* internal carotid artery, *PcomA* posterior communicating artery, *ACA* anterior cerebral artery, *AcomA* anterior communicating artery, *MCA* middle cerebral artery, *PCA* posterior cerebral artery, *SCA* superior cerebellar artery, *PICA* posterior inferior cerebellar artery, *misc.* miscellaneous

### Endovascular procedures

Treatments of the 77 aneurysms were carried out electively over a total of 80 interventions. Flow diversion with the p48 MW device was the primary treatment strategy in 56 (72.7%) aneurysms. The target lesion was a remnant after microsurgical clipping (MSC) in 3 (3.9%) cases (all located at the anterior communicating artery), whereas in 14 (18.2%) cases treatment with the p48 MW device was performed for remnants after previous endovascular coil occlusion of the target aneurysm. Trapping of a persistent posterior communicating artery (PcomA) aneurysm (after previous treatment with flow diversion via the ICA) with a p48 MW device deployed in the posterior cerebral artery (PCA) was carried out in two (2.6%) cases. Excessive foreshortening of the deployed device leading to insufficient coverage of the aneurysmal neck was encountered in 3 (3.9%) cases. No technical issues were encountered in the remaining cases. Good wall apposition was achieved in all cases and no balloon-remodeling was needed in any of the performed procedures.

### Procedural and generic complications

**Fig. 1:**
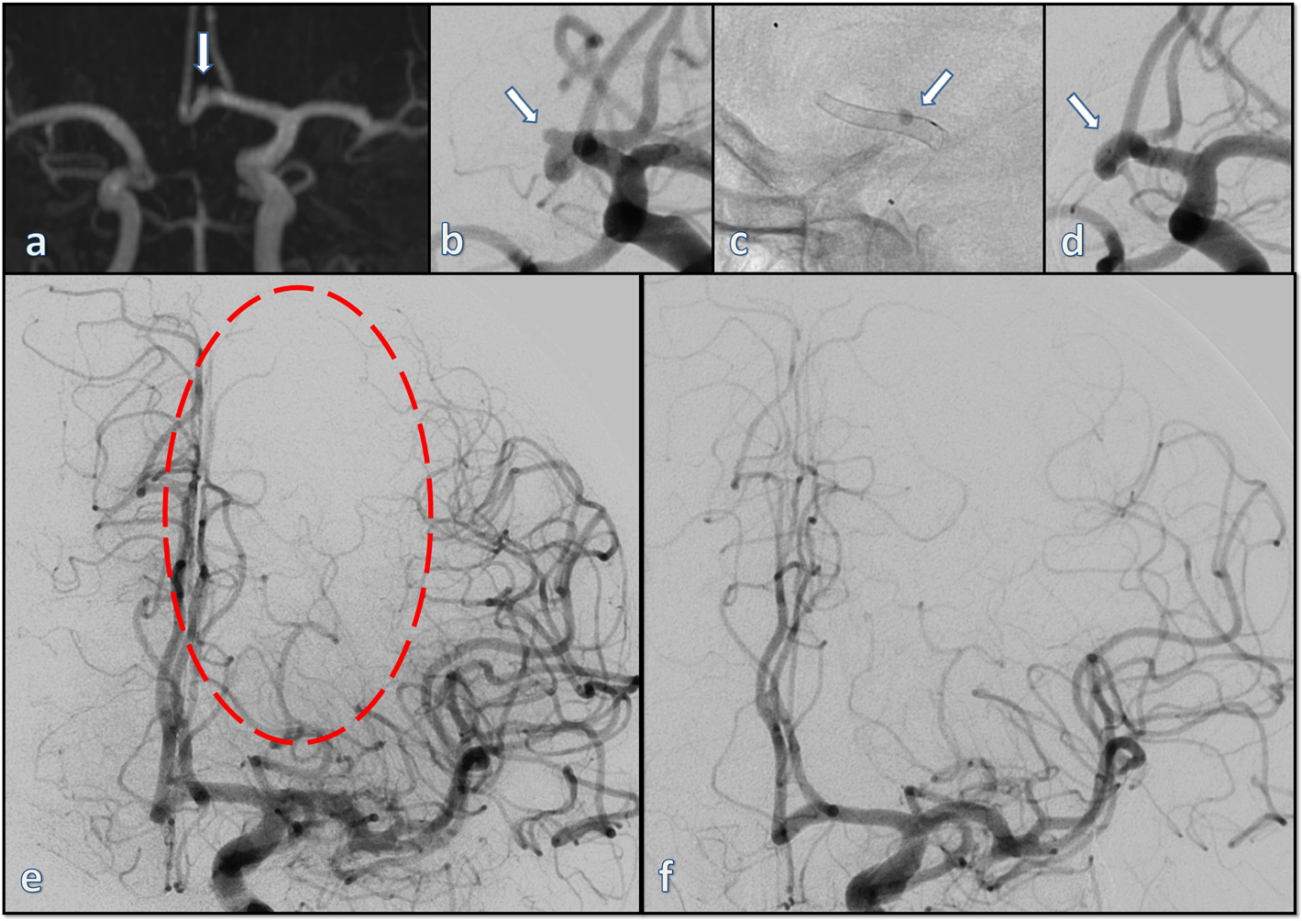
This small, incidental aneurysm of the anterior communicating artery (**a** and **b**) was discovered in the work-up of vertigo. Due to the aplasia of the right A1 segment and closer contact of the aneurysm to the right A2 segment, the p48 MW device was deployed from the right A2 into the left A1 segments (**c**) thereby covering the left A2 segment with subsequent sluggish flow and delayed filling of the left A2 territory (**e**). The patient remained, however, asymptomatic and the aneurysm was completely occluded on follow-up (**d**) with preserved filling of the left A2 segment (**f**). **a** time of flight MR-angiography; **b** working projection; **c** p48 MW device in situ with marked stagnation in the aneurysm; **d** first angiographic follow-up with complete occlusion of the aneurysm; **e** final run in AP projection after deployment of the p48 MW demonstrating the slow filling of the left A2 territory; **f** the angiographic follow-up in AP projection demonstrating the preserved flow in the left A2 despite the initial delayed filling

Taking all adverse events - both temporary and permanent and both symptomatic and asymptomatic - into account, a total of 12 (15%) complications were recorded in this series including 10 (12.5%) peri-procedural (within 24 h), 1 (1.3%) early post-procedural (within 30 days) and one (1.3%) late events (beyond 30 days). Specifically there were three cases (3.8%) of asymptomatic sulcal subarachnoid hemorrhages, three cases (3.8%) of asymptomatic thromboembolic events, two cases (2.5%) of transient neurological deficit, one case (1.3%) of a symptomatic minor ischemia, one case (1.3%) of an asymptomatic sluggish perfusion of a covered anterior cerebral artery (ACA) (Fig. 1), one case (1.3%) of gastrointestinal hemorrhage and one case (1.3%) of sudden death due to apnea in a patient with a known Chiari I malformation, without intracranial hemorrhage or cerebral ischemia. No events were recorded in the remaining 68 (85%) procedures performed in this series.

### Clinical and angiographic follow-ups

**Fig. 2:**
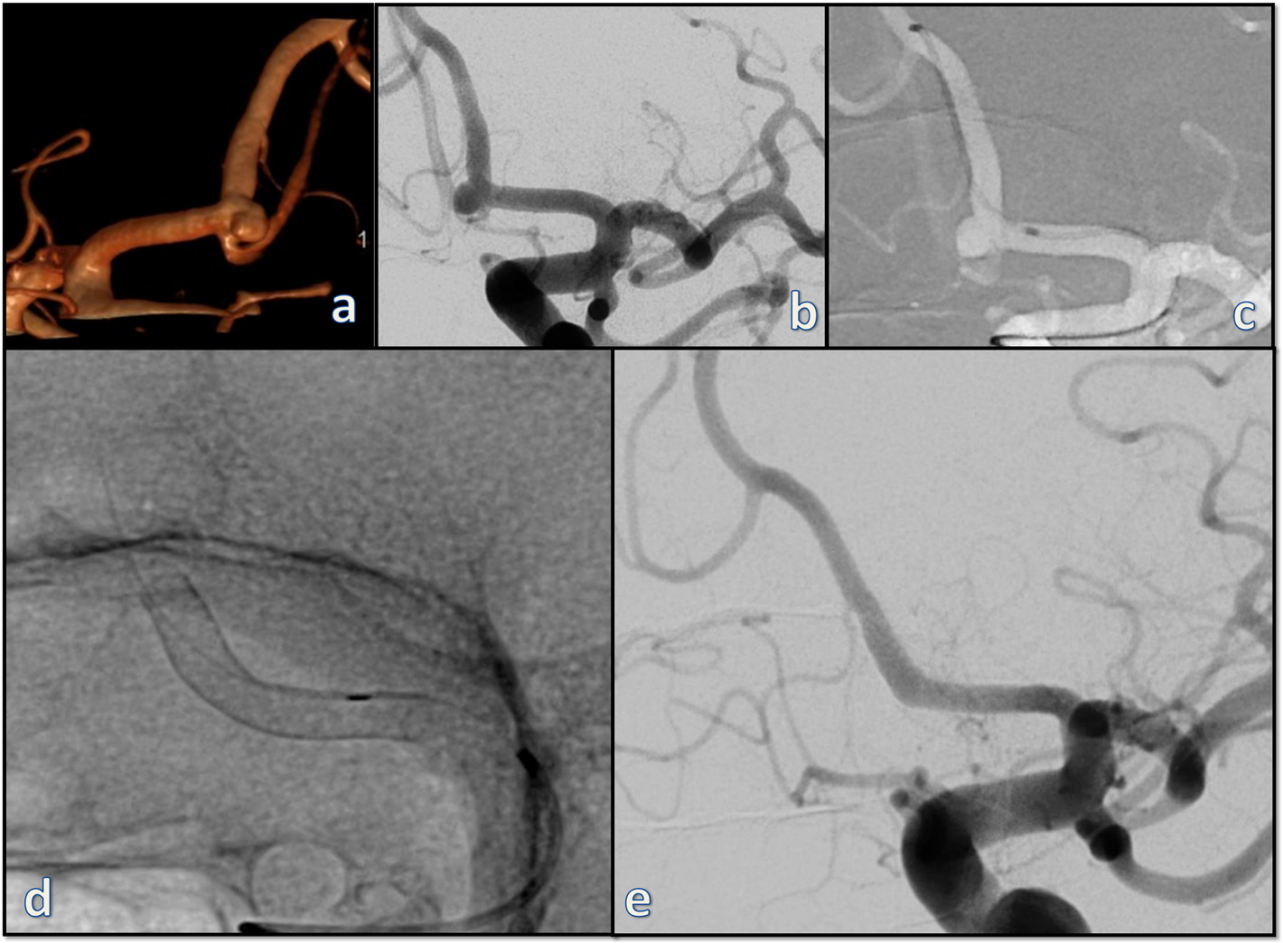
A representative case for treatment of a small, broad-based aneurysm of the left anterior cerebral artery at A1/2 junction with a single p48 MW device and complete obliteration of the aneurysm on the first follow-up. **a** 3D volume rendering of the rotational angiography; **b** working projection; **c** navigation of a 0,021″ microcatheter into the A2 segment; **d** the p48 MW device in situ; **e** first angiographic follow-up

Apart from the aforementioned one case of symptomatic minor ischemia case and one case of sudden death due to apnea, no shifts in the mRS in comparison to the pre-interventional condition have been observed in the remaining 72 (97.3%) patients included in this series. Angiographic follow-up studies were available for 70 (94.6%) patients and 72 (93.5%) aneurysms. The first angiographic follow-up was performed after a mean of 5.1 ± 2.6 months (median 5 months, range 1.2–17 months). Second and third angiographic follow-ups were available for 48 (62.3%) and 14 (18.9%) aneurysms after a mean of 11.2 ± 2.4 and 18.8 ± 4.4 months, respectively. Complete occlusion of the treated aneurysm was demonstrated on the first angiographic follow-up in 27 (37.5%) cases (Fig. 2). A small remnant was observed in 13 (18.1%) cases adding up to an adequate occlusion rate of 55.6% on first follow-up. Twenty-three (71.9%) of the cases with insufficient occlusion on first follow-up had further controls. The occlusion rates are summarized in Table 2. Considering the latest available angiographic studies for all 70 patients who presented to follow-up regardless of its timing, complete and adequate occlusions were observed in 36 (50%) and 46 (63.9%) cases, respectively. Asymptomatic instent-stenosis was observed on the angiographic follow-ups in two (2.6%) cases. There were no cases of side branch occlusions in this series.

**Table 2:** Summary of the results of the available angiographic follow-up studies

FU latency (months)	1st Follow-up (5.1)	2nd Follow-up (11.2)	3th Follow-up (18.8)
**Nr. of cases (%)**	72 (93.5)	48 (62.3)	14 (18.9)
**Complete occlusion (%)**	27 (37.5%)	23 (47.9)	8 (57.1)
**Minor remnant (%)**	13 (18.1%)	8 (16.7)	2 (14.3)
**Major remnant (%)**	16 (22.2)	13 (27.1)	4 (28.6)
**Unchanged (%)**	16 (22.2)	4 (8.3)	0 (0)
**Adequate occlusion**	40 (55.6)	31 (64.6)	10 (71.4)

## Discussion

**Fig. 3:**
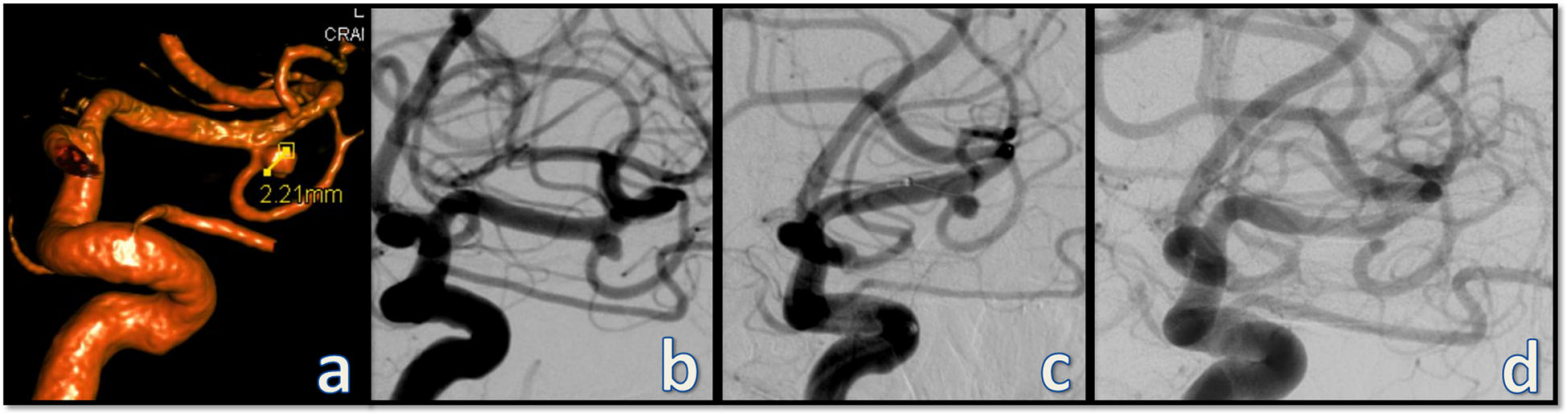
A case of a small, incidental aneurysm of the left middle cerebral artery discovered during the work-up of a transient neurological deficit. The aneurysm is located at the bifurcation of the M1 segment into a dominant superior and a smaller inferior trunk (**a** and **b**). Due to the relatively wide base and intimate relation to the inferior trunk, a single p48 MW device deployed from the superior trunk into the M1 segment covering the aneurysm and the inferior trunk was considered the most appropriate approach. The procedure was carried out uneventfully and the patient was kept on dual antiplatelet therapy for one year. The most recent follow-up angiography (**d**) performed after one year of the treatment shows complete obliteration of the aneurysm and unchanged morphology of the covered inferior trunk. **a** 3D volume rendering of the rotational angiography; **b** working projection demonstrating the intimate anatomic relation of the aneurysm to the inferior trunk; **c** deployment of the p48 MW device; **d** the one-year follow-up

This study represents the largest clinical experience yet with the p48 MW device based on the treatment of 77 saccular intracranial aneurysms. In this study we included only aneurysms treated on elective basis to achieve a homogenous population and eliminate confounding factors that may limit the interpretation of the clinical and radiologic outcomes. The majority of the treated aneurysms were located in the anterior circulation adding up to 84.4% with M1 Segment of the MCA and anterior communicated artery complex being the most common locations. All the treated aneurysms were small (mean diameter 3.5 ± 2.4 mm) and broad based. Flow diversion with the p48 MW device was associated with very low complication rate in this study with only one case of permanent morbidity and one case of procedure-unrelated mortality. The efficacy on the other hand was moderate with adequate occlusion of approximately half of the treated aneurysms on the first angiographic follow-up. The reduced occlusion rate in comparison to first generation of FDS can be hypothesized by the reduced metal density in this low profile device and to some extend by the prolonged DAPT in our institution (DAPT for 1 year followed by monotherapy with ASA indefinitely), which may have led to delayed intra-aneurysmal thrombosis and reduced rate of complete occlusion on the early follow-ups. These two factors may also explain the extremely low rate of instent-stenosis and side-vessel occlusions in this series (2.6% and 0%, respectively) which can be a good trade-off in perforator-rich segments such as the M1 and the basilar arteries or when the aneurysmal anatomy requires the coverage of a major second order vessel such as the A2 or M2 branches (Figs. 1 and 3).

The potential of FDS for the treatment of aneurysms distal to the terminal ICA was explored prior to the availability of such dedicated low profile devices. A Canadian retrospective review of 12 cases treated with the Pipeline Embolic Device for vessels ≤3 mm between 2008 and 2013 reported 92% rate of complete or near-complete occlusion with 8% complication rate (Martin et al., 2015). Cagnazzo et al. reported high efficacy of FDS for the treatment of distal ACA aneurysms but with a 13% rate of complications with permanent sequale (Cagnazzo et al., 2018). A multicohort study from 3 centers involving 46 patients and 46 distal aneurysms, the use of the PED or FRED resulted in nearly 80% rate of complete or near-complete occlusion after a mean follow-up of 13 months with a complications rate of 4.3% resulting form 2 cases of perforator ischemia (Ravindran et al., 2018). A recent multicenter analysis of a relatively large cohort of distal aneurysms involving the second segments of the anterior, middle and posterior cerebral arteries demonstrated a high efficacy rate of flow diversion in these distal and challenging configuration (Primiani et al., 2019). The authors reported a procedural complication rate of 7.7% and 95% rate of good clinical outcome among 60 patients with available clinical follow-ups. Two systematic reviews and meta-analyses reported the feasibility and high efficacy of FDS for the treatment of aneurysms on small or distal vessels but with non-negligible complication rate (Cagnazzo et al., 2019; Yan et al., 2018). The results of these systematic reviews are, however, limited by the selection and publication bias of the included studies. Despite the low quality of the currently available evidence, the use of FDS seems to be associated with high rates of complete and permanent occlusion in small and distal vessels that are comparable to the previously reported results in larger and more proximal vessels (Brinjikji et al., 2013; Kallmes et al., 2017). A major technical limitation in small and distant vessels, however, is the navigability of the delivery device into the intended parent vessel as most commercially available FDS are only compatible with a 0.027″ microcatheter.

The reported experience with the p48 MW Flow Modulation Device is still very limited (Bhogal et al., 2019; Bhogal et al., 2020; Aguilar-Perez et al., 2020). The Buenos Aires experience with the uncoated version based on 25 patients with 25 aneurysms reported an adequate occlusion rate of 79.2% and a procedure-related complication rate of 4% with no permanent morbidity (Bhogal et al., 2019). The cohort of the study was, however, heterogeneous including 16% acutely ruptured aneurysms and 20% non-saccular aneurysms. The p48_HPC is a modified version of the p48 MW device with glycan based hydrophilic polymer coating to reduce the thrombogenicity and is approved for use with a single antiplatelet therapy (SAPT) at the operator discretion. Currently, there is only one clinical report of the use of the p48_HPC under SAPT with Prasugrel in a small series of five patients. The authors reported one hemorrhagic complication and no thromboembolic complication (Bhogal et al., 2020). Our experience with the modified version for the treatment of acutely ruptured aneurysms under single antiplatelet medication was reported in a separate paper (Aguilar-Perez et al., 2020).

Beside the p48 MW, there are currently two commercially available low profile FDS: the Flow Re-Direction Endoluminal Device (FRED Jr., Microvention, Tustin, CA) and the Silk/Silk Vista Baby (Balt, Montmorency, France). The FRED Jr. is a dual-layer nitinol braided flow diverter dedicated for the treatment of vessels of less than 3 mm diameter. The inner layer is braided with 36 wires, whereas the outer layer is braided with 16 wires. The radiopacity is provided by two interwoven tantalum wires and four platinum markers at each end. The FRED Jr. fits through a 0.021″ microcatheter and the recommended vessel diameter is 2–3 mm. In one observational, multicenter study involving 47 aneurysms in 42 patients the FRED Jr. was successfully delivered into the intended vessel in all cases (Möhlenbruch et al., 2017). The overall complication rate was 7% resulting in 2.4% permanent morbidity. Complete or near-complete occlusion was observed in 78% and 100% of the follow-ups at 6 and 12 months, respectively based on follow-ups of 57% and 23% of total population, respectively. A smaller, single-center report of 15 patients treated with FRED Jr. reported no procedure-related morbidities or mortalities and a complete occlusion of 87% on the most recent angiographic follow-up (Rautio et al., 2019). Another single-center report of 12 patients harboring 15 aneurysms at or distal to the circle of Willis reported also no procedure-related morbidities or mortalities. Follow-ups were available for 66.7%% of the aneurysms with complete occlusion rate of 80% (Sivasankar et al., 2019). The Silk Vista Baby is a single-layer flow diverter braided from 48 nitinol wires with an inner platinum core. It is currently the only commercially available FDS compatible with a 0.017″ microcatheter (Headway 17, Microvention). A single-center experience with this device included 25 patients and 27 aneurysms with a parent artery diameter ranging from 1.8–3.5 mm, including 5 cases of distal ICA aneurysms, reported no technical or clinical complications related to the treatment and the rate of complete occlusion on the first follow-up at 3 months was 62.9% (Schob et al., 2019). A larger, multicenter series including 41 consecutive patients and 43 aneurysms reported 12.2% rate of intraprocedural complications with no clinical sequale and no shift in the mRS score compared to the admission status (Martínez-Galdámez et al., 2019). Follow-ups were not available in this series but the immediate occlusion rate was 18.6%.

## Limitations

This report is a single-center, retrospective and single-arm design study with its inherent limitations. All the included aneurysms were unruptured or not acutely ruptured limiting the interpretation of the results to the treatment of elective cases. The lack of long-term angiographic follow-up studies may have undermined the efficacy of the device as aneurysmal occlusion after flow diversion is progressive. The relatively large study cohort, on the other hand, allows more reliable conclusions regarding the safely and efficacy due to the extensive experience of the involved operators with this new device.

## Conclusion

Elective endovascular treatment of small, saccular aneurysms with the p48 MW flow modulation device is safe and feasible and is associated with moderate efficacy on early follow-up. The very favorable safety profile and very low rate of side-vessel modification or occlusion make this device a valid treatment option for aneurysms located at major side-branch or perforator-rich vessel segments. Further technical modification with anti-thrombogenicity coating may widely broaden the spectrum of treatable lesions to include acutely ruptured aneurysms.

## Data Availability

The entire data as well as case documentations are available in anonymous form from the senior author upon reasonable request.
